# The Success Rate of Pediatric In-Hospital Cardiopulmonary Resuscitation in Ahvaz Training Hospitals

**DOI:** 10.1155/2016/9648140

**Published:** 2016-05-16

**Authors:** Shideh Assar, Mohsen Husseinzadeh, Abdul Hussein Nikravesh, Hannaneh Davoodzadeh

**Affiliations:** ^1^Pediatric Department, Golestan Hospital, Ahvaz Jundishapur University of Medical Sciences, Iran; ^2^Pediatric Department, Abuzar Hospital, Ahvaz Jundishapur University of Medical Sciences, Iran; ^3^Golestan Hospital, Ahvaz Jundishapur University of Medical Sciences, Iran; ^4^Department of Anesthesiology, Ahvaz Jundishapur University of Medical Sciences, Ahvaz, Iran; ^5^Medical Research Center, Jundishapur Health Development Co., Tehran, Iran

## Abstract

*Research Objective*. This study determined the outcome of cardiopulmonary resuscitation (CPR) after in-hospital cardiac arrest and factors influencing it in two training hospitals in Ahvaz.* Method*. Patients hospitalized in the pediatric wards and exposed to CPR during hospital stay were included in the study (September 2013 to May 2014). The primary outcome of CPR was assumed to be the return of spontaneous circulation (ROSC) and the secondary outcome was assumed to be survival to discharge. The neurological outcome of survivors was assessed using the Pediatric Cerebral Performance Category (PCPC) method.* Results*. Of the 279 study participants, 138 patients (49.4%) showed ROSC, 81 patients (29%) survived for 24 hours after the CPR, and 33 patients (11.8%) survived to discharge. Of the surviving patients, 16 (48.5%) had favorable neurological outcome. The resuscitation during holidays resulted in fewer ROSC. Multivariate analysis showed that longer CPR duration, CPR by junior residents, growth deficiency, and prearrest vasoactive drug infusion were associated with decreased survival to discharge (*p* < 0.05). Infants and patients with respiratory disease had higher survival rates.* Conclusion*. The rate of successful CPR in our study was lower than rates reported by developed countries. However, factors influencing the outcome of CPR were similar. These results reflect the necessity of paying more attention to pediatric CPR training, postresuscitation conditions, and expansion of intensive care facilities.

## 1. Introduction

Pediatric cardiorespiratory arrest is an extremely stressful incident for pediatricians and children's families [1]. Over 200,000 in-hospital cardiac arrest instances with survival rates of less than 20% occur in the United States, annually [2]. The most common cause of cardiac arrest in adults is cardiovascular ischemic disease [3, 4]. Unlike adults, the most important causes of cardiac arrest in children are not primary cardiac causes [5, 6]. In these age groups, cardiac arrest is mostly caused by advanced respiratory failure and shock, which is known as asphyxial arrest. Asphyxia starts from a variable time following hypoxia, hypercapnia, bradycardia, and hypotension and ultimately leads to cardiac arrest. The other pediatric cardiac arrest mechanism includes cardiac causes such as cardiomyopathy, arrhythmia, and genetic cardiac diseases which account for about 15 to 25% of cardiorespiratory arrests [7]. At the early years of application of the new CPR method, only 9% of children would survive following cardiorespiratory arrest while the figure increased slightly in the first twenty years of application of this method [7]. In 1988, other important measures were also added to the treatment of pediatric cardiorespiratory arrest [8]. As a result, the resuscitation methods were divided into the groups of preliminary and advanced in-hospital and out-of-hospital techniques [8]. In the study by the American Heart Association, which was carried out for printing the new resuscitation guideline, it was found out that in 10 years (from 2000 to 2009) the rate of in-hospital resuscitation success to discharge increased from 14% to 43% whereas it is still below 10% for out-of-hospital resuscitation. As a result, new CPR strategies aim at improving in-hospital resuscitation [9]. The rate of survival to discharge after in-hospital cardiac arrest is higher among children than among adults (47% versus 18%). It shall be mentioned that the level of shockable arrhythmia during cardiac arrest (VT-VF) in children is 1/4 times that of adults whereas the success of resuscitation during asystole in children is 2.5 times that of adults [10]. As a result, the aim of this study was to determine the outcome of CPR after in-hospital cardiac arrest and relevant factors in children hospitalized in two major training hospitals of Ahvaz from September 2013 to May 2014 (Golestan Hospital and Abuzar Hospital).

## 2. Method

The permission of the ethical committee of Ahvaz Jundishapur University of Medical Sciences was obtained. The present study was a cross-sectional descriptive-analytical study which caused no physical or financial damage to the patients. Patients' information remained confidential and all patients signed informed consent. The research inclusion criteria allowed for the selection of patients who were below 18 years and were hospitalized in the pediatric units of Abuzar and Golestan training hospitals of Ahvaz and their CPR procedures were done by residents. Study exclusion criteria were out-of-hospital cardiorespiratory arrest; duration of resuscitation for less than one minute; age over 18; and resuscitation by hospital staff other than residents. Pediatric residents, who had attended preliminary and advanced pediatric CPR workshops according to the global resuscitation guidelines and protocols (revised in 2010) and had received training on infants or children, were employed in the study and took responsibility for the research. A questionnaire was arranged and completed for resuscitated patients by residents involved in CPR. According to inclusion and exclusion criteria, 279 cases were finally included in the research. The questionnaire included questions about the demographic information of patients (first name, last name, file number, age, gender, and weight), resuscitation information (the unit and time in which arrest occurred, administered medicines, preresuscitation condition of the patient, and resuscitation success), and information about the educational level. If a patient was exposed to more than one resuscitation, only information about the first resuscitation was considered. After CPR, patients who survived to the time of discharge were selected and their neurological condition was examined at the time of discharge by the researchers. PCPC score was completed for all the patients. This score includes 6 cognitive states (1 = normal, 2 = poor disability, 3 = average disability, 4 = extreme disability, 5 = coma or vegetative state, and 6 = death). The 6 aforementioned states are classified into good, average, and poor neurological states. A good cerebral performance is associated with PCPC scores of 1 and 2 at the time of discharge or no changes from the prehospitalization neurologic state. Finally, for the purpose of information analysis, the descriptive statistics methods such as the frequency distribution table, diagrams, and central indices (such as mean, dispersion, and standard deviation) were used to describe the study variables. The data was analyzed using the following statistical tests: *t*-test, chi-square test, and one-way analysis of variance. The significance level of the above tests was assumed to be below 0.05 and data was analyzed using SPSS version 20.

## 3. Results

In the course of the research, 392 CPR attempts were done. After reviewing the questionnaires, 113 were omitted based on the exclusion criteria and 279 cases, which matched the research objectives, were studied. Of the 279 resuscitated patients, 138 patients (49.4%) experienced ROSC after 20 minutes with or without administration of drugs (and were considered the primary survival cases). The highest success rate of primary survival (50 patients or 36.3%) was observed in the PICU whereas the lowest rate of successful primary survival (22 patients or 15.9%) was observed in other units. Of the 138 patients who experienced successful primary survival (ROSC), 81 cases survived 24 hours after the resuscitation (secondary survival). Therefore, patients with secondary survival constituted 29% of all the CPR cases and 59% of the primary survival population. The highest number of patients who survived to 24 hours was in the PICU (30 patients, 37%) whereas the lowest number was in the NICU and other wards (14 patients (17.3%) and 14 patients (17.3%), resp.). Global survival to discharge was 11.8% (23.9% of the patients who achieved ROSC). The lowest rate of survival to discharge was observed in NICU (8.1% or 7 patients) (Table 1). According to PCPC score system, neurological condition of 16 of 33 patients (48.5%) who survived to discharge was assessed to be good (with PCPC score of 1 to 3 or unchanged prehospitalization score). Overall, 5.7% of all resuscitated patients had good neurological state at discharge. The highest level of discharge in good neurological conditions was observed in NICU (5.8% or 5 patients) whereas the lowest level was observed in PICU (3.1% or 3 patients). Only 2 patients had a PCPC score of 1.

### 3.1. Location of Arrest Occurrence

Of the 279 resuscitation patients, most patients were in PICU (33.7% or 94 patients) and the remaining patients (13.6% or 38 patients) were in other hospital units. Considering the number of patients hospitalized in this period, the total rate of resuscitation in the population hospitalized in both hospitals was 4.05%. The highest rate of resuscitation in the population hospitalized in the study period was seen in NICU (24.4%). The ratio of resuscitated patients to the number of hospitalized patients was 19.5% and 4.7% in PICU and emergency room, respectively. No significant relationship was observed between the location of incidence of arrest and survival.

### 3.2. Preresuscitation State

Before the occurrence of arrest, 188 patients (67.3%) were exposed to cardiac monitoring and had the oxygen saturation marker. Moreover, 157 patients (56.2%) were also intubated while 132 patients (47.3%) constantly received infusions of vasoactive medications (such as dopamine).

Regarding primary survival, patients with unsuccessful resuscitation were significantly more than the group with successful resuscitation in receiving infusion of vasoactive medicine and intubation before resuscitation (Table 2).

### 3.3. Intraresuscitation Conditions

Table 2 shows the number of patients who received epinephrine, sodium bicarbonate, normal saline, and intravenous calcium, respectively, during resuscitation. The relationship of an intraresuscitation increase in epinephrine dosage and administration of calcium and bicarbonate was significantly related to unsuccessful resuscitation (*p* < 0.05).

Only 29 patients (10.4%) were exposed to cardioversion. However, no significant relationship was observed between the application of shock and the outcome of resuscitation (Table 2).

### 3.4. Initial Cardiac Rhythm

Upon examination of the cardiac rhythm of 279 resuscitated patients, bradycardia and asystole, which were observed in 201 cases (72%), had the highest frequency whereas shockable rhythms of ventricular tachycardia and ventricular fibrillation were observed in 12 cases (4.3%).

The highest frequencies of shockable rhythms were seen in PICU (8 cases or 67%). No relationship was observed between initial cardiac rhythm and the outcome of resuscitation (*p* = 0.91).

### 3.5. Primary Diseases

Assessment of the prearrest underlying diseases in resuscitated patients revealed that the highest frequencies of arrests were caused by respiratory diseases and infectious diseases in 93 (33.3%) and 52 (18.6%) patients, respectively. Metabolic and genetic conditions were observed in 12 patients (4.3%) and had the lowest frequency. The rate of survival to discharge in patients with respiratory problems was significantly higher than in those with other underlying diseases (*p* = 0.045). A significant relationship was observed between renal diseases and the unsuccessful outcome of resuscitation (*p* = 0.03) (Table 3).

### 3.6. Age

The average age of patients was 34.9 months (from a minimum of 1 day to a maximum of 17 years). Moreover, 35.1% of patients were aged below one month and 31.9% were infants. The rates of 24-hour survival after resuscitation and survival to discharge in the infants were significantly higher than those of other age groups (Table 4).

A relationship was found to exist between the neurologic outcome at the time of discharge and age in the infants group (*p* = 0.03). However, in the other groups, no relationship was observed (Table 4).

### 3.7. Duration of Resuscitation

**Figure 1:**
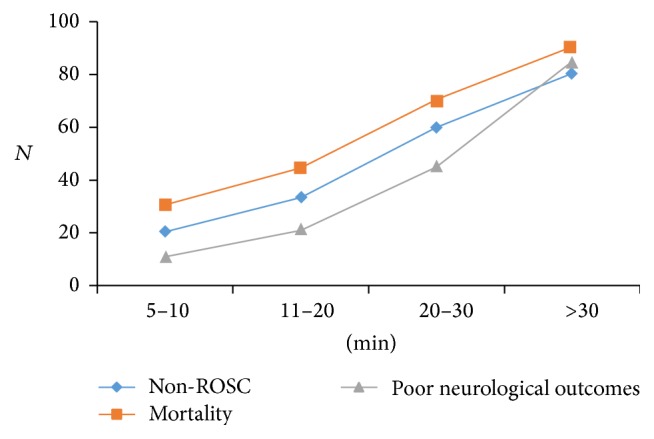
Duration of resuscitation.

The average duration of resuscitation in patients was 39.5 minutes (from a minimum of 5 to a maximum of 55 minutes with a median duration of 20.5 minutes). The final success of resuscitation was remarkably higher in patients who went through shorter resuscitation as compared to the patients with longer resuscitation periods (26.5 ± 15.2 versus 15.1 ± 8.7). A longer resuscitation period was accompanied by worse neurological outcomes especially in patients with resuscitation periods of more than 20 minutes (*p* = 0.001). Of the 94 patients who had a resuscitation time of more than 30 minutes, only 7 patients (2.5%) survived to discharge and only one of the 7 patients was released in good neurological conditions (Figure 1).

### 3.8. Time of Cardiac Arrest Occurrence

**Figure 2:**
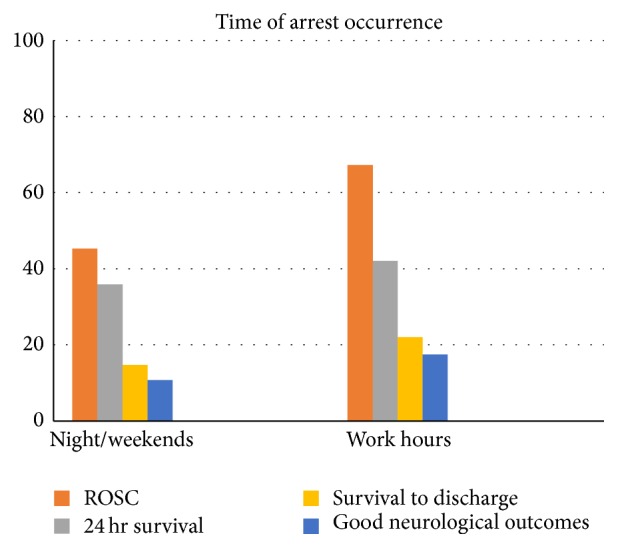
Time of cardiac arrest occurrence.

In the course of this study, 125 patients (44.8%) were resuscitated at nighttime (between 20:00 and 8:00 the next morning). In addition, 89 (31.9%) and 65 (23.3%) patients were resuscitated in the evening (between 14:00 and 20:00) and in the morning (between 8:00 and 14:00). Of these patients, 75 (26.9%) cases were resuscitated during holidays (Figure 2).

Patients who were resuscitated at night or on weekends demonstrated lower rates of ROSC compared to other patients (45.3% versus 67.2%). However, no significant differences were observed between the discharge outcome (14.7% versus 22%) and neurologic outcome (10.7% versus 17.5%) patients who were resuscitated on working days and daytime.

The average interval between hospitalization and the occurrence of arrest was 26 days for the successful resuscitation group and 10 days in the unsuccessful resuscitation group. No significant relationship was observed between the duration of hospitalization before the arrest and the success of resuscitation (*p* = 0.06) (Figure 2).

### 3.9. Gender and Growth Status

Of the 279 resuscitated patients, 156 were male and 123 were female. No significant relationship was seen between gender and final outcome of resuscitation (Table 5).

The average weight of patients was also 9.2 kg (a minimum of 1.2 and a maximum of 73 kg). No relationship was observed between the weight of patients and the outcome of resuscitation.

After examining the weight of patients against the existing weight-age and weight-height diagrams, the growth of patients was measured. According to the results, 12 (4.3%) patients were overweight and 44 (15.8%) were underweight. A significant relationship was observed between underweight and unsuccessful resuscitation (*p* = 0.04) (Table 5).

### 3.10. Education Level of the Resuscitation Resident

**Figure 3:**
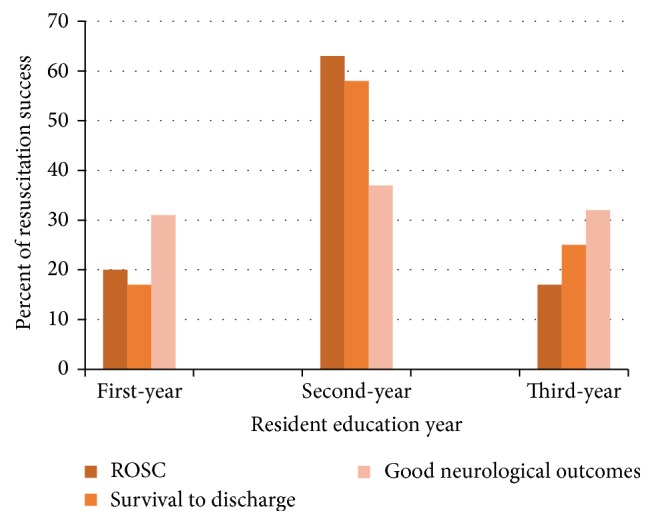
Education level of the resuscitation resident.

**Figure 4:**
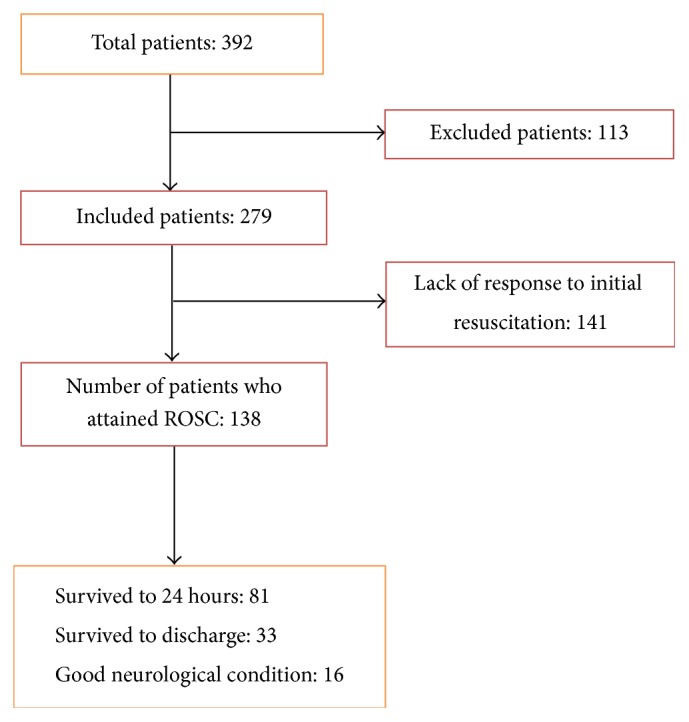
Total patients.

Of the 279 resuscitated patients, second-year residents handled 150 resuscitation procedures (53.7%) which was the highest number of resuscitation procedures followed by first-year residents who carried out 86 resuscitation procedures (30.8%). Finally, the third-year residents also conducted 43 resuscitation procedures (15.4%). Investigating the relationship between the education level of resuscitation residents and the outcome of resuscitation revealed that there was a significant relationship between unsuccessful resuscitation outcome (unsuccessful primary survival) and resuscitation by a first-year resident (*p* = 0.03). However, no significant relationship was seen between the neurological outcome of patients and the education level of the resuscitation residents (Figure 3). All of this result was shown in Figure 4 briefly.

## 4. Discussion and Conclusion

As reported in Section 3, of the 279 patients studied in this research, 138 patients (49.4%) experienced ROSC. Moreover, 29% of patients lived 24 hours after resuscitation and 11.8% survived to discharge. Of the patients who survived to discharge, 48.5% were released in good neurological conditions. According to the information published by the National Registry of Cardiopulmonary Resuscitation (NRCPR), about 43 to 64% of resuscitated patients experience ROSC while 36% of patients live for 24 hours after resuscitation and 25–33% survive to discharge. Of the patients who live to discharge, about 3/4 are released in good neurological conditions. In the study by Wu et al. in Taiwan, 55% of patients lived for 24 hours after resuscitation and 21% survived to discharge. More than 55% of the latter patients were released in good neurological conditions [11]. In the research by López-Herce et al. in Spain, 41% of patients survived to discharge and approximately 78% were discharged in a good neurological condition [12]. The rates of success in both of the aforementioned studies were higher compared to the present research. Concerning secondary survival, which reflects the postresuscitation conditions of patients, it should be noted that ECMO was used in both the Taiwanese and the Spanish studies for patients with advanced cardiopulmonary failure. Therefore, new methods contribute to the prognosis of resuscitation. In another study, del Castillo et al. stated that 74% of patients experienced ROSC whereas 41% survived to discharge. Of the patients who survived to discharge, 77.9% were released in a good neurological condition [13].

### 4.1. Primary Disease

Moreover, Ortmann et al. carried out a study in Philadelphia on patients who were classified into the following groups: the cardiothoracic surgery group, cardiomedical group, and noncardiac group. It was reported that the rate of 24-hour survival in the aforementioned first, second, and third groups was 60%, 42%, and 37%, respectively. The number of patients discharged in good neurological condition in the cardiothoracic surgery, cardiomedical, and noncardiac groups was 77%, 72%, and 70%, respectively [14]. However, in the current study, the arrest etiology and background of patients were also considered. In this regard, it is worth noting that the survival to discharge among renal patients was lower than among other patients, which can be ascribed to the complex and chronic condition of most of them. Among patients with underlying diseases, cases with respiratory conditions experienced successful resuscitation and demonstrated a higher rate of survival to discharge compared to patients with other underlying diseases. However, it is worth mentioning that this finding requires further investigations due to the high importance of function of the respiratory system after resuscitation. In the present study, the highest incidence of arrest was observed in respiratory cases followed by patients with infectious diseases. But, in the Taiwanese research, the highest incidence was among cardiac cases followed by hematologic-oncologic and neurologic patients [11]. In addition, del Castillo et al. reported that the minimum mortality was observed in cardiac patients whereas the highest level of mortality was seen in oncology patients [13]. Rodríguez-Núñez et al. found the highest level of mortality in patients with sepsis histories [15]. These findings show the difference in the prevalence of diseases in different societies. Nevertheless, it should be noted that, in the study period, respiratory diseases accounted for the largest number of hospitalized patients.

### 4.2. Location of Arrest Occurrence

NRCPR reports that about 86% of cardiac arrests occur in the ICU among patients under monitoring whereas only 14% of cardiac arrests occur in general units (that lack monitoring) [10]. In the present study, about 65% of in-hospital arrests occurred in ICU which shows that arrest mostly occurred in high-risk patients under study. However, the resulting figure (65%) is still lower than the ones reported by NRCPR (86%) and the Taiwanese study (86.2%) [11, 16]. This finding reflects the necessity of paying more attention to the identification of high-risk patients who need to be transferred to ICU.

### 4.3. Pre-CPR Monitoring

In this study, about 32% of patients were not exposed to cardiac monitoring before the arrest whereas in the NRCPR report only 14% of patients had this problem. This finding again reflects the need for the development of healthcare facilities [16].

### 4.4. Time of Cardiac Arrest Occurrence

According to a study by the American Heart Association which was conducted for the 2010 Guideline, the rate of survival at nighttime and on weekends is lower than that of weekdays. Moreover, the rate of survival also declines after the administration of vasopressin before the arrest [17]. In this study, with an increase in the number of intraresuscitation epinephrine dosages and the duration of resuscitation, mortality increased. The level of mortality also increased on weekends. According to the report by NRCPR, the increase in the number of patients who live 24 hours after resuscitation is the result of the presence of residents and fellowships following resuscitation [16]. The investigations in the present study also showed that, in the mornings and weekdays, when residents and attendants were present in the hospital, the success of resuscitation and ROSC was higher. However, no significant difference was observed between the 24-hour survival and the final outcome of patients in the aforementioned days/times and on weekends. Wu et al. reported from Taiwan that the rate of primary survival (ROSC) at nights or on weekends is lower than on weekdays [11]. This finding complies with the results of the present study. It can be perhaps explained by the minimum presence of personnel on weekends, multiplicity of duties, and the fatigue caused by weekday works as the investigations by Herlitz et al. showed that the decrease in the number of medical staff is significantly related to the number of in-hospital arrests with unsuccessful resuscitation [18]. Moreover, the study by Jayaram et al. showed that the rate of survival at night and on weekends was 26% and 25.2%, respectively [19]. In this research, the postresuscitation rate of survival to discharge among infants (one month to one year) was higher than that of other age groups (*p* < 0.01).

### 4.5. Age

Moreover, the neurologic outcome of patients in the infant group was also better (*p* = 0.03). The same finding was reported by NRCPR [20]. In the study by Meaney et al. which was carried out on 464 patients with cardiac arrest, the overall rate of survival to discharge was 22%. The rate of survival in neonates, infants, and children was 27%, 36%, and 19%, respectively. The latter figure declined to 16% in adolescents. However, the highest level of positive neurological outcome was seen in infants (20%) whereas the lowest level was seen in children over 8 years (11%) [21]. In the study by del Castillo et al., the rate of mortality in 1-month-old to 12-month-old infants was 57.1% [13]. However, in the study by Jayaram et al., the rate of survival in neonates and infants was 30.4% and 30.8%, respectively [19].

### 4.6. Intraresuscitation Conditions

Similar to the NRCPR study, the resuscitation investigations in the Spanish and Taiwanese studies indicated that there was a significant relationship between the dosage of vasoactive medicines administered before arrest and increased mortality [11, 13, 22]. This can be explained by the fact that these drugs are basically used for patients in fatal conditions. Moreover, the results of the current study showed that the relationship of administration of calcium and bicarbonate and more than two dosages of epinephrine with unsuccessful resuscitation is significant. This is also shown in other studies [10, 20, 23], especially the one by Warren et al. that examined 20909 patients from 505 hospitals. In the aforementioned study, it was reported that a decrease in the number of prescriptions of epinephrine leads to an increase in the survival of patients [2].

### 4.7. Duration of Resuscitation

The present study showed that increased duration of resuscitation results in unsuccessful resuscitation. Of the 94 patients who were resuscitated in more than 30 minutes, only 7 patients (2.5%) survived to discharge and only one of the 7 patients was released in a good neurological condition. None of the patients with resuscitation duration of more than 40 minutes had successful resuscitation. Similar findings were obtained from other studies. In their investigations, López-Herce et al. found out that when the duration of resuscitation is more than 20 minutes, the final mortality rate is expected to increase to 78%. Moreover, when the duration of resuscitation is increased to more than 60 minutes, the mortality rate reaches 100% [20]. Rodríguez-Núñez et al. showed that the resuscitation duration of between 10 and 19 minutes is associated with a mortality rate of 72.7% whereas the resuscitation duration of more than 20 minutes is associated with 100% mortality rate [15]. Moreover, Rodríguez-Núñez et al. also stated that 90% of patients who were resuscitated in less than 10 minutes experienced ROSC. Of these patients, 60% survived to discharge from hospital [24]. del Castillo et al. also reported that when resuscitation takes longer than 30 minutes, the mortality rate is 91.7% whereas, in resuscitation procedures that lasted less than 5 minutes, the rate of mortality is 35.6% [13].

### 4.8. Heart Rhythm

It was found out that bradycardia is the most common initial cardiac rhythm which was similar to the findings of other studies [10, 11, 18]. It was also indicated that children respond better to asystole than do the adults. Unlike the adults, the shockable rhythms in children do not leave a significant effect on the final outcome.

### 4.9. Education Level of the Resuscitation Resident

It was indicated that resuscitation procedures carried out by first-year residents lead to the worst outcomes. It seems that the curriculum for the resuscitation workshop of the first-year residents should be revised so that the residents would be tested at the end of the workshop and their scientific and practical abilities for resuscitation would be ensured. In the study by Dr. Soltani et al. on the pediatric residents of Tehran University, it was indicated that special training on PALS improves the performance and attitude of residents [25].

In the end, it is worth mentioning that although the lower success rate of resuscitation in the centers studied in this research demonstrates the need for more concern for training residents and all the medical staff on resuscitation and revising the training courses, factors such as the cultural level of families (which often leads to late referral to medical centers) and the large volume of patients attending the referral centers (which does not match the number of service providers) should also be considered among the influencing factors. Planning and appropriate education are required to address these problems.

In sum, it is found out that factors influencing the resuscitation outcome in our study were similar to those reported by other valid articles. According to the results of this study and the findings of other research, more intense CPR training is recommended in new residents. In addition, it is recommended to use senior experienced residents to the possible extent and also employ special personnel to assist the resuscitation team during holidays and at nighttime.

## Figures and Tables

**Table 1:** Location of occurrence of arrest.

	PICU *N* (%)	NICU *N* (%)	Emergency ward *N* (%)	Other wards *N* (%)	*p* value	Total
Number of patients	94 (33.7%)	89 (31.8%)	60 (21.5%)	36 (13%)		**279**
Primary outcome	50 (36.3%)	33 (23.9%)	33 (23.9%)	22 (15.9%)	0.67	**138**
Survival to 24 h	30 (37%)	14 (17.3%)	23 (28.4%)	14 (17.3%)	0.51	**81**
Survival to discharge	12 (36.4%)	7 (21.2%)	9 (27.3%)	5 (15.1%)	0.81	**33**
Good neurological outcome	3 (18.7%)	5 (31.3%)	5 (31.3%)	3 (18.7%)	0.63	**16**

**Table 2:** Pre- and intraresuscitation conditions.

	Survivor (*n*: 33)	Nonsurvivor (*n*: 246)	*p* value
*Pre-CPR condition*			
Monitoring	19 (57.5%)	169 (68.7%)	**0.201**
Endotracheal intubation	4 (12.1%)	153 (62.2%)	**0.01**
Continuous infusion of vasoactive drugs	2 (6%)	130 (52.8%)	**0.015**
*CPR management*			
Epinephrine > 0.02 mg/kg	2 (6%)	107 (43.5%)	**0.022**
Calcium injection	4 (12.1%)	105 (42.6%)	**0.04**
Bicarbonate injection	8 (24.2%)	174 (70.7%)	**0.001**
Normal saline infusion	10 (30.3%)	106 (43.4%)	**0.162**
Cardioversion	2 (6%)	27 (10.9%)	**0.068**

**Table 3:** Primary disease.

Primary disease	*n* (%)	Primary outcome	Survival to 24 h	Survival to discharge	Good neurological outcome	*p* value (survival to discharge)
(i) Respiratory	93 (33.3%)	57 (41.3%)	38 (46.9%)	17 (51.5%)	7 (43.7%)	**0.045**
(ii) Infection (nonrespiratory)	52 (18.6%)	30 (21.7%)	16 (19.7%)	6 (18.1%)	4 (18.7%)	**0.067**
(iii) Cardiac	43 (15.5%)	22 (15.9%)	12 (14.8%)	5 (15.1%)	3 (18.7%)	**0.51**
(iv) GI/hepatic	31 (11.1%)	11 (7.9%)	6 (7.5%)	2 (6.1%)	1 (6.2%)	**0.81**
(v) Neurologic	30 (10.7%)	7 (5%)	4 (5%)	2 (6.1%)	1 (6.2%)	**0.055**
(vi) Nephrologic	18 (6.5%)	7 (5%)	2 (2.5%)	0	0	**0.03**
(vii) Metabolic/genetic	12 (4.3%)	4 (2.9)	3 (3.7%)	1 (3%)	0	**0.61**

**Table 4:** Age.

	<1 month	1–12 months	1–8 years	9–18 years	*p* value
	*N* (%)	*N* (%)	*N* (%)	*N* (%)	
Number of patients	98 (35.1%)	89 (32%)	60 (21.5%)	32 (11.4%)	
Primary outcome	42 (30.4%)	57 (41.3%)	26 (18.9%)	13 (9.4%)	**0.67**
Survival to 24 h	21 (25.9%)	38 (46.9%)	16 (19.7%)	6 (7.5%)	**0.31**
Survival to discharge	8 (24.3%)	17 (51.5%)	6 (18.1%)	2 (6.1%)	**0.01**
Good neurological outcome	5 (31.2%)	7 (43.7%)	4 (25.1%)	0	**0.03**

**Table 5:** Gender and growth status.

	Survivor (*n*: 33)	Nonsurvivor (*n*: 246)	*p* value
*Gender*			
Male	18 (54.5%)	138 (56%)	**0.07**
Female	15 (45.5%)	108 (44%)	
*Growth pattern*			
Normal	23 (69.7%)	200 (81.3%)	**0.051**
Overweight	3 (9%)	9 (3.65%)	**0.057**
Underweight	7 (21.2%)	37 (15%)	**0.04**

## Additional Points

Since the present study was a cross-sectional study, the effect of the study season on the findings is evident. That is to say, due to the prevalence of infectious and respiratory diseases in cold seasons, the rate of mortality among children by these diseases is relatively increased. Particularly, in order to identify prevalent underlying diseases for arrest, it is necessary to conduct long-term studies that last about 1 or 2 years. On the other hand, due to the importance of resuscitation, the physician should fully pay attention to the resuscitation and survival of the patient. Therefore, the relevant form is completed after the patient is in a stable state and, as a result, a few things might be forgotten or the information on the form might not fully comply with the reality due to the fear of legal or disciplinary problems.
